# Innovative payer engagement strategies: will the convergence lead to better value creation in personalized medicine?

**DOI:** 10.1007/s13167-017-0078-6

**Published:** 2017-02-13

**Authors:** Ildar Akhmetov, Rostyslav V. Bubnov

**Affiliations:** 1 Strategic Market Intelligence Department, Unicorn, P.O. Box 91, Zhytomyr, 10020 Ukraine; 2 Clinical Hospital “Pheophania” of State Affairs Department, Zabolotny Str., 21, Kyiv, 03680 Ukraine; 3 Zabolotny Institute of Microbiology and Virology, National Academy of Sciences of Ukraine, Zabolotny Str., 154, Kyiv, 03680 Ukraine

**Keywords:** Predictive preventive personalized medicine, Payer engagement strategy, Value creation, Market access, Reimbursement

## Abstract

**Background:**

As reimbursement authorities are gaining greater power to influence the prescription behavior of physicians, it remains critical for life science companies focusing on personalized medicine to develop “tailor-made” payer engagement strategies to secure reimbursement and assure timely patient access to their innovative products. Depending on the types of such engagement, pharmaceutical and diagnostic companies may benefit by obtaining access to medical and pharmacy claims data, getting invaluable upfront inputs on evidence requirements and clinical trial design, and strengthening trust by payers, therefore avoiding uncertainties with regards to pricing, reimbursement, and research and development reinvestment. This article aims to study the evolving trend of partnering among two interdependent, yet confronting, stakeholder groups—payers and producers—as well as to identify the most promising payer engagement strategies based on cocreation of value introduced by life science companies in the past few years. We analyzed the recent case studies from both therapeutic and diagnostic realms considered as the “best practices” in payer engagement. The last 5 years were a breakout period for deals between life science companies and reimbursement authorities in the area of personalized medicine with a number of felicitous collaborative practices established already, and many more yet to emerge. We suggest that there are many ways for producers and payers to collaborate throughout the product life cycle—from data exchange and scientific counseling to research collaboration aimed at reducing healthcare costs, addressing adherence issues, and diminishing risks associated with future launches.

**Conclusions:**

The presented case studies provide clear insights on how successful personalized medicine companies customize their state-of-the-art payer engagement strategies to ensure closer proximity with payers and establish longer-term trust-based relationships.

## Background

The emergence of comparative effectiveness research and the shift of the healthcare industry toward novel outcome-based payment models have put diagnostic and pharmaceutical companies in a less convenient, yet propitious, position in personalized (and so-called precision) medicine. On one hand, the need to provide convincing evidence for the superiority of therapeutic, diagnostic, or drug delivery solutions over the existing alternatives has escalated developmental costs and created additional market access complexities. On the other hand, companies that are able to justify the value of their products by demonstrating a measurable differentiation vis-à-vis competitors have a luxury to enjoy high premiums and gain unique competitive advantage to a large extent due to more extensive reimbursement and wider stakeholder acceptance.

Considering heterogeneity of stakeholder needs, diversity of tools to quantify value of products, and breadth of possible clinical and nonclinical evidence, it remains critical for innovative companies with focus on personalized medicine to partner with institutional customers, particularly payers, to create the aligned incentives and support the necessary capabilities to make value-based deals work. Depending on the types of such collaboration, life science companies may benefit by obtaining access to medical and pharmacy claims data, getting invaluable upfront inputs on evidence requirements and clinical trial design, and strengthening trust by payers, therefore avoiding uncertainties with regards to pricing and reimbursement.

The recent survey conducted among 75 managed care executives in the USA showed that partnering between payers and biopharma becomes a prevailing wind in the industry with more than 70% of respondents recognizing value in the early stage collaboration [1]. Notwithstanding that payers recognize the vitality of pharma data presented upfront, only 31% of the payers claim to take part in phase III clinical trials, and less than a quarter involve in the earlier phase testing. The reasons for such discrepancy are multifold: lack of trust in manufacturers, pharma’s underestimation of the importance of early stakeholder engagement, and even more so the inability of the industry to navigate the two topmost payer challenges—compliance and cost containment.

The aforementioned signifies that there is a clear gap in the way the industry and payers interact, making it exceedingly difficult for both parties to realize the full potential of their convergence. However, it also implies the tremendous opportunities for pharmaceutical and diagnostic companies focusing on precision medicine to address this gap by designing and implementing more sophisticated precompetitive programs involving payers in the mature and fast-growing markets.

This paper aims to study the evolving trend for partnering among the two interdependent, yet confronting, stakeholder groups—payers and producers—as well as to identify the most promising payer engagement strategies based on cocreation of value introduced by companies with focus on personalized medicine in the past few years.

## The growing influence of payers and innovation crisis

The dramatic spike in healthcare costs over time has put an enormous pressure on payers to toughen the reimbursement criteria for pharmaceutical and diagnostic products in the developed and emerging markets. Exerting greater influence on the global healthcare ecosystem, payers (governments, pharmacy benefits managers, public and private insurance plans, and managed care organizations) have set up an unspoken rule that obtaining FDA, EMA, or CE approval no longer provides the life science companies with a privilege to charge premium prices and secure reimbursement.

Today, reimbursement authorities are demanding additional evidence that goes far beyond the customary safety and efficacy data, incorporating relative cost-effectiveness and budget impact analyses in their formulary decisions. As a result, innovative companies continue suffering from intense drop-offs in positive decisions for new product reimbursement.

Thus, out of 102 cancer drug indications appraised by the National Institute for Health and Care Excellence (NICE) between 2007 and 2014, only 47 (46%) were fully or partially recommended for NHS commissioning [2]. Similar numbers were observed in Germany in the period of 2011–2014, where Institute for Quality and Efficiency in Health Care (IQWIG) determined that 50% of the assessed new drugs offered “no additional benefit” vis-à-vis comparators [3]. Moreover, substantial private and public reimbursement rejections were evidenced among molecular diagnostic and therapeutic companies in the USA and Australia particularly due to the inability to justify value-for-money [4, 5].

Recognizing the growing influence of payers and accounting for the direct correlation of insurance premiums with the escalating medicinal costs, large medical societies, such as American Society of Clinical Oncology (ASCO) and American Heart Association (AHA), are now considering on incorporating costs or “financial toxicity” as another important perspective in the guidelines [6, 7]. The rise in healthcare expenditure has also created much anxiety amid physicians in the countries where retirement benefit or other financial incentives are affected by overprescribing [8]. With pressure from public and private payers, the cost-sensitive prescribers become less and less influential in their clinical decisions often giving preferences to cheaper and less effective options over highly effective therapeutics and diagnostics that are not covered by health plans.

For example, in the Netherlands, 75% of the total drugs (including Herceptin®) in about 1/3 of the Dutch hospitals were uncovered by the healthcare insurance in the mid-2000s, leading to unequal access to highly effective and costly medicines [9]. And it is only the 7-year Herculean effort of the two patient advocacy groups—the Dutch Breast Cancer Association and the Dutch Federation of Cancer Patient Organizations—that changed the status quo of Herceptin®, even though the final impact on the cost control and reimbursement procedures in the Netherlands were still not achieved.

While the economic expediency and ethics associated with payer-guided healthcare provision are not that straightforward and are always subject to lengthy debates, it is quite obvious and particularly critical that a significant undervaluation of diagnostics and therapeutics by means of severe pricing and reimbursement restrictions leads to an underincentivized innovation. According to Thomson Reuters and Deloitte joint survey, the cost of bringing a new medicine from discovery to launch has escalated from USD 1.1 billion in 2010 to USD 1.3 billion in 2013 (+18%). Moreover, the burden of late stage failures was estimated to deprive the top 12 pharma companies of USD 243 billion in 2010–2013 [10].

As innovative companies prioritize their portfolios based on commercial expectations, health insurance reimbursement levels are the key economic signals for innovators to determine whether the likely market for a new diagnostic or therapeutic will be large enough to substantiate costly investments. Hence, bearing high marginal costs and getting low incentives from payers, today, profit-driven innovative companies become more rigid in revising their pipeline assets in terms of reimbursement attractiveness, frequently limiting patient access to high-quality products for fear of a coverage fiasco.

Thus, in the early 2000s, several large pharmaceutical companies have abandoned antibiotics research and investment prioritizing drugs in other classes, such as cancer and heart disease. This resulted in the fact that resistance to existing medicines continued to increase, while the number of new antibiotics receiving FDA approval declined [11, 12]. In 2014, the problem was addressed by the President’s Council of Advisors on Science and Technology that assembled miscellaneous stakeholders to create new aligned incentives facilitating research and development (R&D) in antibiotics [13].

All of these suggest that the current approach to healthcare coverage is imbalanced and, in light of the growing influence of payers, it is crucial to revise the way “innovation” is defined and valuated, outlining unambiguous incentives for the life science companies to continue reinvesting into R&D.

## “Per aspera ad astra”—innovative payer engagement strategies

**Fig. 1:**
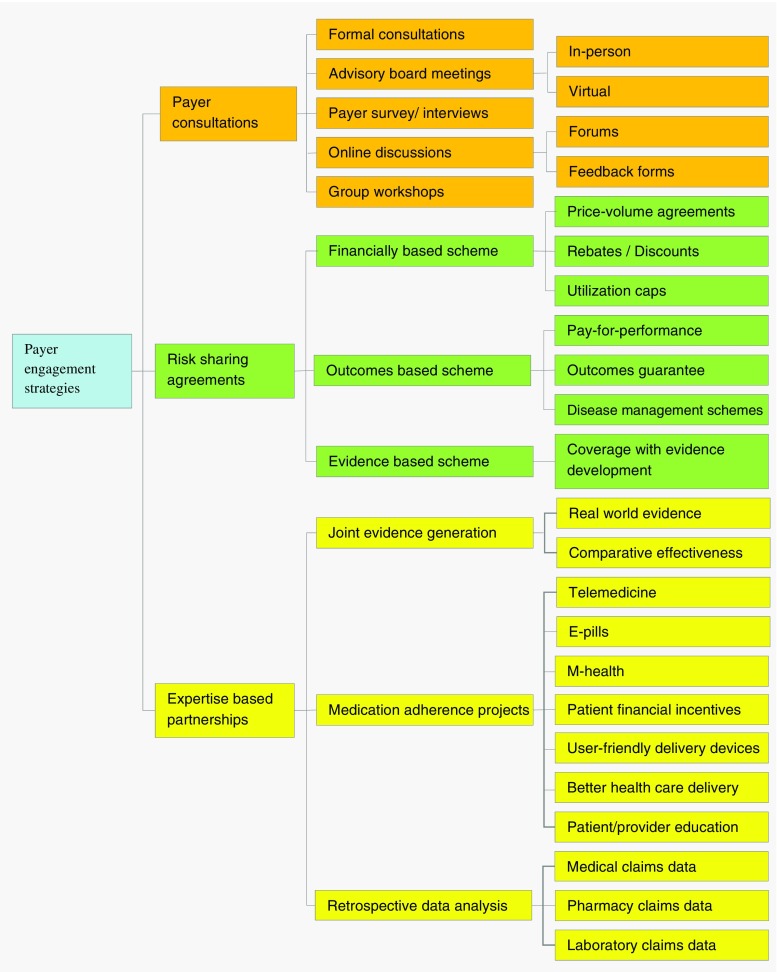
Examples of innovative payer engagement strategies in personalized medicine

In the past several years, the life science companies have been responsive to the new payer role by means of expanding their market access and regional sales teams to target the specific reimbursement accounts, collecting more data on payer “pain points” and decision-making processes, customizing core value dossiers to the local payer needs, and allocating substantial funds on localized real-world evidence studies. Although important, these strategies provide companies with only incremental benefits, and do not allow the necessary proximity with reimbursement authorities. Hence, to achieve such proximity, companies should have more open dialogues and tighter collaboration with payers based on trust and cocreation of value (Fig. 1).

Reimbursement authorities, uncertain of what manufacturers have in their pipelines, generally appreciate early dialogues with the life science companies to be more informed about impending launches and the associated additional budget loads. In response, companies are able to address their questions and create more clarity on what they can do to make the pipeline and marketed therapeutics more attractive from a payer perspective. Yet, despite of market access and commercial teams creating a myriad of brilliant ideas on how to partner with reimbursement authorities, there are always ethical and regulatory constraints about going beyond the conventional unit price-based relationships and co-pays. One of the prime concerns of the healthcare stakeholders regarding the industry–payer partnerships is transparency.

To address this issue and to harmonize early dialogues between HTA agencies and the producers of health technologies, recently, there has been launched a new initiative in Europe called “Shaping European Early Dialogue” (SEED) [14]. This pilot consortium, led by the French Haute Autorité de Santé (HAS) and financed by the European Commission (EC), is currently covering 10 projects (7 for therapeutics and 3 for medical devices), which will facilitate the development of methodological guidelines on the early stage payer–producer interaction.

A number of legislative acts incentivizing industry–payer convergence have also appeared at a national level. For example, in the UK, the Health and Social Care Act of 2012 has introduced a framework to encourage collaboration between the National Health Service and life science companies aimed at facilitating innovation and healthcare delivery [15].

The early dialogues between manufacturer and reimbursement authorities are particularly important in light of the existing inconsistencies in the way “innovation” is defined by both parties. It frequently occurs that producers consider “me-too” medicines as innovative because of their distinct manufacturing process and differences in the mechanism of action vis-à-vis the standard of care. On the contrary, reimbursement authorities pay more attention to the overall comparative effect of a therapeutic versus the associated costs, and little are they interested in the surrogate endpoints or technological peculiarities of an innovative product.

According to Bach (2009), prices of the majority of anticancer therapeutics are escalating with greater velocity than the health benefits associated with them, making each treatment progress less cost-effective than the previous one [16]. Having no ultimate way to assess the true value of innovation, payers are guided by a simple principle that innovation without a strong differentiation should not be included in their drug formularies, while a unique therapeutic or diagnostic solution with greater relative effectiveness targeting a high unmet need is more likely to see the reimbursement green light.

Yet, the borderline between “strong” and “weak” differentiation varies greatly between HTA and reimbursement authorities, making it literally impossible to adequately quantify innovation. For example, the recent study conducted by Droeschel et al. showed that of the 20 identical oncology therapies evaluated by French and English HTA bodies, 17 have been recommended by HAS, and only 4 received positive decisions by NICE [17].

As suggested by Fig. 1, apart from early dialogues with companies, payers recognize substantial value in almost all kinds of research initiatives related to cost mitigation—from participation in real-world evidence studies to engagement in a comparative effectiveness research. For example, in 2009–2011, Medco Health Solutions (acquired by Express Scripts) has launched a large head-to-head study of Plavix® (clopidogrel) and Effient® (prasugrel) stretching out over 2 years to identify the strata of heart patients, who were not able to metabolize clopidogrel and required to be switched to prasugrel based on their genetic makeup [18]. Thus, being able to direct patients to less expensive generic versions of clopidogrel, the pharmacy benefit manager acquired more confidence that it had achieved comparable care at a significantly reduced cost.

Along the same lines, in 2011 AstraZeneca has signed an agreement with WellPoint’s HealthCore to establish a 4-year evidence of development partnership generating real-world data via prospective and retrospective observational studies on the most effective and economical treatments in chronic and other diseases [19]. As a part of this deal, companies were to analyze electronic medical records, review claims information, and conduct patient surveys.

Another attractive area for life science companies to collaborate with reimbursement authorities is poor medication adherence, which is perhaps the hottest issue faced by payers these days along with cost containment. Hence, by leveraging innovative patient-centered solutions (e.g., financial incentives, telemedicine, e-pills, m-health, user-friendly drug delivery devices, and improving healthcare delivery) companies are able to win trust and create greater value for payers.

The Merck–Cigna collaboration on Januvia® and Janumet® is one of the successful industry–payer partnership examples resulting in 87% improved patient adherence to therapy, in which Merck introduced large discounts to patients who followed the medication regime as prescribed, and Cigna lowered co-pay levels for adhering patients [8]. Another case of WellDoc’s BlueStar also perfectly illustrates how a mobile-integrated therapy coupled with electronic health records enables adults with type 2 diabetes to achieve statistically significant improvements in HbA1c level, enhance patient satisfaction and win reimbursement [20, 21].

Establishing risk-sharing agreements (also, “managed entry agreements,” “patient access schemes,” “RSAs,” “MEAs,” and “PAS”) is another broadly utilized approach to engage with payers, for such arrangements offer the potential for earlier access to new therapeutics and diagnostics by linking reimbursement levels to real-world performance or use of a product. RSAs aim at sharing financial risks due to uncertainty related to the introduction of new technologies [22].

There are three major groups of RSAs [23]:

1. Financially based schemes define producer’s contributions to the cost of a therapeutic or diagnostic based on financial thresholds, and not linking coverage to health outcomes (e.g., price–volume agreements, rebates, discounts, and utilization caps);
2. Outcome-based schemes are performance-based agreements, in which health-related quality of life predetermines pricing and reimbursement levels (e.g., pay-for-performance, outcomes guarantee, and disease management schemes);
3. Evidence-based schemes (also, “coverage with evidence development” or “CED”) are a category of industry–payer deals, in which a company is granted temporary reimbursement in exchange for generation of supplementary real-world evidence with the subsequent revision of coverage conditions based on the collected evidence.

According to Ferrario and Kanavos, Belgium, England, Sweden, and the Netherlands had 133 active RSAs as of 2012, with English payers giving preferences to discounts and free doses; Dutch prioritizing CED; Swedes considering CED, monitoring utilization and compliance with restrictions via observational studies; and Belgians equally using all of the aforementioned practices [24]. The utilization of RSAs is also gaining momentum in the Asia-Pacific region with 98 schemes observed in Australia, 5 in New Zealand and 3 in South Korea [23]. The emergence of such innovative approaches to market access is an imperative milestone in formulating new generation payer–producer agreements based on open dialogue, cooperation, and multi-stakeholder value creation.

Among other premises for the industry and payers to collaborate is the fact that reimbursement authorities are understaffed, budget-constrained and bogged down in their routine, often underperforming in the areas of disease management, data analysis, as well as patient and provider education [25]. Hence, there are tremendous opportunities for these stakeholders to partner filling the aforementioned gaps on account of a disease-specific expertise and resources.

For instance, last year, Novo Nordisk has launched an initiative called cities changing diabetes, where it partnered with a number of large private insurers, like United Healthcare, Blue Cross Blue Shield, Aetna, and Cigna, to tackle the problem of urban diabetes in the USA, analyzing key factors that drive the global pandemic and utilizing that knowledge to shape real-world solutions [26]. Likewise, in 2011, Pfizer teamed up with Humana to bring together data and scientific information to create a better comprehension of patient needs and drug effectiveness [27].

To sum up, there are numerous opportunities for producers and reimbursement authorities to collaborate based on the unmet payer needs, including the necessity to reduce healthcare costs, poor adherence, high uncertainty and risks associated with future launches, and lack of specific expertise and resources. Successful life science companies that are able to customize their state-of-the-art payer engagement strategies to these needs may achieve closer proximity with payers and establish longer-term trust-based relationships.

## Case studies

The last 5 years were a breakout period for deals between life science companies and reimbursement authorities in the area of personalized medicine with a number of felicitous collaborative practices established already, and many more yet to emerge. Below are several case studies from both therapeutic and diagnostic realms, considered as the “best practices” in payer engagement:

### “Roche–Swiss Re” collaboration in China

Roche is a global healthcare company developing innovative therapeutic and diagnostic products in the areas with unmet medical needs. As of 2014, three out of ten best-selling blockbuster medicines in oncology were developed and commercialized by Roche [28].

In 2010, the company lined up with Swiss Re to strengthen its presence in the Chinese market by introducing risk-sharing deals to local insurers. Since direct commerce of foreign retail insurance was prohibited by law in China, Roche and Swiss Re came up with a revolutionary idea to start selling reinsurance, and thus convincing Chinese insurers to offer coverage for high-quality cancer therapeutics. As a part of this collaboration, Roche provides Swiss Re and local insurers with marketing support and statistical data on different types of cancer, while Swiss Re provides Chinese insurance companies with reinsurance and technical support on the product design and pricing [29].

Within just 2 years, the Roche–Swiss Re collaboration in China allowed the local insurance policy holders, including CPIC Life, to achieve their first milestone—selling insurance to 6 million customers—thus facilitating access of a large number of cancer patients to innovative treatments, like trastuzumab [30, 31].

### “VitrOmics–CZ–CPCT” partnership in the Netherlands

VitrOmics Healthcare Holding is an organization specialized in personalized medicine focusing on the development of diagnostic products to measure kinase and nuclear receptor activities, based on the PamChip® platform created by its daughter company PamGene.

In the early 2012, the company launched a private–public collaborative initiative engaging with the Dutch health insurance provider CZ and the Centre for Personalized Cancer Treatment with the goal to address the problem of overtreatment in breast cancer, providing much more selective therapy to a stratified subpopulation of patients than stipulated by the usual clinical practice [32].

As a result, the triad was awarded a value-based healthcare prize under the patronage of an honorary chairman Michael E. Porter, establishing a benchmark for “good practice” in industry–payer partnerships based on cocreation of value and trust. This initiative has also prepared the ground for two subsequent trials in lung and esophageal cancer.

### “Eli Lilly–Humana” initiative in the USA

Eli Lilly is a global biopharmaceutical company with primary focus on noncommunicable chronic diseases and nearly a century of experience in diabetes research.

In 2013, the company announced joint research collaboration with a large healthcare insurance company, Humana, in order to conduct a retrospective analysis of medical, pharmacy, and laboratory claims data with the primary objective—to identify modifiable characteristics of diabetic patients associated with increased healthcare costs, as well as to study therapeutic interventions affecting patients’ adherence and outcomes [33].

This data will enable Eli Lilly design more targeted therapeutic interventions and disease management programs in the future, and assist Humana in addressing adherence and cost issues in diabetes. Similar research initiatives were also introduced by Pfizer, Merck, AstraZeneca, and other companies, tightly collaborating with leading health insurance providers [27].

### “MolecularMD–Medco” partnership in the USA

MolecularMD is a manufacturer of molecular diagnostic tests for oncology applications, including a qRT-PCR assay to measure BCR-ABL levels prior to initiation of chronic myeloid leukemia (CML) therapy. The Philadelphia chromosome (BCR-ABL) is found in 95% of CML patients and is currently considered to be the primary cause of the disease [34, 35].

In 2011, the US leading pharmacy benefit manager Medco Health Solutions has launched a program titled “DNA Direct® by Medco™” in collaboration with MolecularMD to identify patients eligible for treatment, thereby reducing costs for treating patients that are not responsive [35].

This collaborative patient-centered initiative led to a significant reduction in healthcare costs for Medco, increased market share and revenues of MolecularMD, improved decision-making of physicians, and enhanced the quality of care of patients. Apart from the CML program, Medco has also signed partnerships with other life science companies to identify patients eligible for warfarin, clopidogrel, tamoxifen, abacavir, and maraviroc.

### “Nuo Therapeutics–CMS” risk-sharing deal in the USA

Nuo Therapeutics is a biomedical company developing and commercializing individualized regenerative wound-healing therapies. The company’s flagship product is Aurix®, an autologous hematogel used to treat diabetic foot, pressure, and venous leg ulcers.

In 2010, facing a national noncoverage determination on blood-derived products for chronic nonhealing wounds, Nuo Therapeutics has initiated a dialogue with the Centers for Medicare & Medicaid Services (CMS) on how to abolish this national mandate for Aurix®, considering the robust safety and effectiveness profile of its product [36]. In 2011, the mandate for Aurix® was removed, yet the CMS concluded that although promising, the provided data was not sufficient to validate the product’s generalizability to the Medicare population, hence, additional real-world evidence would have been required for Aurix® to be included in the positive reimbursement list. In 2014–2015, after the approval of protocols for launching the three prospective randomized effectiveness trials, the positive CED decision was granted to Nuo Therapeutics [37].

## Global pricing policies

Currently, we observe the debate on global pricing policies in particular to develop essential medicines and ensure its affordability and availability in the longer term for a consistent supply in developing countries, like antiretrovirals, artemisinin combination therapies, drug-resistant tuberculosis medicines, liposomal amphotericin B (for visceral leishmaniasis), and pneumococcal vaccines, and to promote policies that “de-link” the financing of R&D from the price of medicines [38].

The role of international alliances, not-for-profit enterprises, is constantly increasing, e.g., the contribution of Global Alliance for Tuberculosis Drug Development (TB Alliance) to a public–private partnership, that draws upon the resources of both private and public institutions to help address this urgent health need aims to stop the spread of tuberculosis by developing new, faster-acting, and affordable tuberculosis drugs [39]. The Global Fund [40] works to support programs of prevention, treatment, and care for AIDS, tuberculosis, and malaria and to strengthen health systems. Eligible countries can receive an upfront allocation, which provides improved predictability of funding. Starting from the country’s national strategic plan, stakeholders participate in an ongoing and inclusive country dialogue.

The several examples of the so-called “philanthrocapitalism” that include private initiatives/efforts of Bill and Melinda Gates [41] and Chan Zuckerberg Initiative (CZI) [42] demonstrate fair success and might become more important player to help fighting challenging inequalities in healthcare funding in the future.

### Horizon 2020 and personalizing health and care

During the first 4 years of Horizon 2020 (work programs for 2014/2015 and 2016/2017), the EU research funding program Horizon 2020 (2014–2020) and in particular the Commission’s proposal for health, demographic change, and wellbeing challenge [43] will invest more than €2 billion in calls for proposals or actions considered as an investment in better health for all, aims to support the development of new, safer, and more effective interventions to test and demonstrate new models and tools for health and care delivery and also contributes to the sustainability of health and care systems [43, 44].

Granting schemes of the European Commission, like the largest public–private initiative innovative medicines initiative (IMI) [45] with a total budget of €130 million aiming to speed up the development of better and safer medicines for patients addresses therapy and diagnostic development challenges to develop novel solutions for unmet market needs.

Thus, relevant Horizon 2020 calls like “The development of new methods and measures for improved economic evaluation and efficiency measures in the health sector” focus on economic model development [46]; EU funding for precommercial procurement (PCP) and public procurement of innovative solutions (PPI) projects are available across the different 2016–2017 Horizon 2020 work programs [47] overcoming barriers in precompetitive public procurements of innovative solutions in particular from SMEs.

This case study signifies the importance of early dialogues with payers. Yet, it also demonstrates that although Nuo Therapeutics was rewarded for its persistence and collaborative approach to CMS, much time and financial resources could have been saved, should the company establish the dialogue with the reimbursement authority earlier in the developmental phase.

## Conclusions

Reimbursement authorities, substantially free from regulatory constraints and driven by the escalating healthcare expenditures, are gaining greater power to impact the prescription behavior of physicians. Although the payers’ attempts to contain healthcare costs may have a positive economic effect from a national health service perspective, it may also have a negative clinical affect from an individual patient perspective, due to restricted coverage of effective premium-priced products (direct impact), as well as via underincentivization of innovation (indirect impact).

In the past several years, the life science companies have been responsive to the new payer role by redesigning sales and market access teams, adjusting and localizing their clinical and pharmacoeconomic studies in accordance with the identified payer requirements, as well as conducting more research on payer needs. Considering that these strategies provide pharmaceutical and diagnostic companies with only incremental benefits, producers are advised to have more open dialogues with payers upfront and to establish tighter collaborative initiatives with reimbursement authorities based on trust and cocreation of value.

There are many ways for producers and payers to collaborate throughout the product life cycle—from data exchange and scientific counseling to research collaboration aimed at reducing healthcare costs, addressing adherence issues, and diminishing risks associated with future launches. Successful life science companies that are able to design their state-of-the-art payer engagement strategies around payer needs may achieve closer proximity with these stakeholders and establish longer-term trust-based relationships.

Gazing into the future, scenarios in which payers invest into the life science companies to develop “tailor-made” therapeutic or diagnostic products seem not to be that far off, although the ethical and legal concerns associated with such practices are also likely to emerge. Moreover, we anticipate a greater number of early dialogue initiatives between life science companies and public payers (not only private payers) to arise at the regional, national and international levels. With the emergence of pan-European programs, like SEED, it is also expected that early dialogues with payers will become more transparent and systemized, which will lead to prompter market access of new therapeutic and diagnostic products. These and other topics are subject to our future scientific research.

## Outlook and expert recommendations

Current paper conform with the PPPM objectives summarized in the EPMA White Paper 2012 [48] to support personalized medicine to be widely adopted in clinical set to yield safer, more effective, and less expensive medical interventions [39] and provide stakeholders with evidence of its clinical, cost-effectiveness, and financial viability. Personalized medicine raise investors’ interest and expectations and desires who provide the required capital. Ultimately, the big pharmaceutical industry has been dithering to receive a personalized medicine approach as it was expected that patient stratification would prompt drastically diminished market sizes [48, 49]. However, there is evidence that personalized medicine, based on biomarkers, may demonstrate superior effectiveness that warrants higher pricing. Many new personalized medicines can be expected to be cost-effective because of the increased clinical effect, even if they have premium prices. On the other hand, in many countries, to link reimbursement to cost-effectiveness via clear dialogue between the commercial and scientific groups become a common practice ensuring a “right culture” for promoting truth-seeking rather than progression-seeking behavior [50] for differentiated and reimbursable medicine, including important elements, like market opportunity, scientific understanding and unmet medical needs, evaluating risk, and probability of technical and regulatory success. The recent massive resources devoted to genome-sequencing studies paradoxically provided very little new evidence. However, a better strategy for identifying new treatment options may be to develop methods for analyzing the signaling networks that underlie disease development, progression, and therapeutic [51–53]. National and international granting strategies in PPPM are constantly discussed by European initiative, such as EPMA [54], pilot programs to support prospective healthcare have already been set. Since its very beginning, EPMA is systematically working on the economy of PPPM [4, 44, 48, 52, 54–58].

Potential problems/limitations of implementation personalized medicine approach, which still faces an uncertain future, since being addressing prevention rather than targeted therapy, is more expensive than existing successful preventive interventions [59–61]. Personalized medicine affecting the costs of medical care alters the future of the entire healthcare system and has suggested that medical care costs will be reduced in the future. Another challenge for implementing personalized medicine in low- and middle-income countries (LMICs) [60] is the lack of awareness of needs for personalized medicine among the general population, low educational level (overall literacy rates for many LMICs less than 67%), however this statement might be is stereotyped—e.g., Ukraine is formally LMIC, but demonstrate rather high educational level of population.

We suggest the further studies on the following points:

- the development of new methods and measures for improved economic evaluation and efficiency measures in the health sector to gain advances in increasing value, promote health-seeking behaviors;
- strategic planning for reimbursement, the output of new products on the market and patient access to innovations on all stages—development predictive markers for patients stratification, personalized treatments, and prevention and beyond;
- avoid overusing unnecessary clinical diagnostic, screening, and therapeutical interventions that lack sufficient evidence on efficacy and safety via value-guided approach;
- develop multicriteria models for valuing medicines [62];
- to organize relevant clinical trials to search the evidence, comparative effectiveness research (CER) using population-based, retrospective data can inform assessments of personalized medicine, including implementation of N-of-1 trials [63];
- the development educational programs in compliance with experts in medical fields consider ethical issues to balance involvement of big pharmacology companies
- adaptation of National medical legislation; political support will likewise be expected to help for accomplishing normal repayment amongst suppliers and payers, so that forthcoming consideration can satisfy its guarantee of being the cost-effective medical model to improve national health systems;
- adhere the medical legislation in developing countries to meet requirements of novel and effective models;
- to involve small companies which have higher R&D productivity compared with larger companies [64];
- to develop applicable business models for LMICs for reduction in health disparities between resource-rich countries and LMICs;
- further research is needed to develop the models to boost the reimbursement policy for effective nonpharmacological approaches, like physical therapy, pain management, musculoskeletal ultrasound-guided interventions [65, 66], treatments via probiotics, personalized diets [67], and regenerative medicine, which recently demonstrated high level of efficacy and safety [68];
- consider ethical issues, security, and privacy of medical and financial information [56];
- implementation of personalized approach should be done smoothly via development accessible healthcare and National medical economy and updated legislation for inclusive, innovative and reflective societies [69].

AHA, American Heart Association; ASCO, American Society of Clinical Oncology; CE, Conformité Européenne; CED, Coverage with Evidence Development; CMS, Centers for Medicare & Medicaid Services; EC, European Commission; EMA, European Medicines Agency; FDA, Food and Drug Administration; HAS, Haute Autorité de Santé; HTA, health technology assessment; IQWIG, Institute for Quality and Efficiency in Health Care; NHS, National Health Service; NICE, National Institute for Health and Care Excellence; RSA, risk-sharing agreements; SEED, Shaping European Early Dialogue; LMIC, low- and middle-income countries.
